# DS21, a new noninvasive technology, is effective and safe for screening for prediabetes and diabetes in Chinese population

**DOI:** 10.1186/s12938-020-00823-x

**Published:** 2020-10-14

**Authors:** Xiaopeng Zhu, Jing Tang, Huandong Lin, Xinxia Chang, Mingfeng Xia, Liu Wang, Hongmei Yan, Hua Bian, Xin Gao

**Affiliations:** 1 Department of Endocrinology, Zhongshan Hospital, Fudan University, Shanghai, 200032 China; 2 Fudan Institute for Metabolic Disease, Fudan University, Shanghai, 200032 China; 3 Changqiao Community Health Service Center, Shanghai, 200032 China

**Keywords:** Diabetes, Prediabetes, Impaired glucose regulation, Screening

## Abstract

**Background:**

Screening for prediabetes and asymptomatic diabetes is important for preventing development to an irreversible stage. The current diagnosis of prediabetes and diabetes is based on blood glucose or HbA1c (an invasive method). The aim of this study was to assess the efficacy and safety of DS21, a new noninvasive technology, for noninvasive screening for prediabetes and diabetes.

**Methods:**

A total of 939 subjects were divided into a normal control group (NC, *n* = 308), impaired glucose regulation group (IGR, *n* = 312), and diabetes (DM) group (*n* = 319). All subjects underwent the DS21 test, and mean hands–feet, hand, and feet conductance values were analyzed. The diagnostic accuracy of the conductance value was analyzed by receiver-operating characteristic (ROC) curve.

**Results:**

The conductance values for hands–feet, hands, and feet in the DM and IGR groups were significantly lower than those in the NC group (all *P* < 0.01). The area under the ROC curve  (AUCROC) for distinguishing NC/IGR was highest when using hands–feet conductance values (0.766 [95% confidence interval, CI 0.730, 0.803]). However, the AUCROCs of distinguishing NC/abnormal glucose metabolism (AGM, including IGR+DM), non-diabetes (NDM)/DM, and IGR/DM were highest when using conductance values for hands at 0.782 [95% CI 0.752, 0.812], 0.688 [95% CI 0.653, 0.723] and 0.573 [95% CI 0.528, 0.617], respectively (all *P *< 0.01). Hand conductance of values 75.0 (sensitivity 0.769, specificity 0.660), 77.1 (sensitivity 0.718, specificity 0.695), 68.4 (sensitivity 0.726, specificity 0.555), and 58.1 (sensitivity 0.384, specificity 0.744) were recommended as the screening thresholds for NC/AGM, NC/IGR, NDM/DM, and IGR/DM, respectively. A hand conductance value 66.0 was also recommended to distinguish NC/AGM due to its high sensitivity and high PPV. No adverse events occurred in the test.

**Conclusions:**

DS21 is fast, noninvasive, low cost, reliable and safe, which makes it a feasible device for screening for prediabetes and diabetes, especially in a large population.

## Background

Diabetes has become a serious public health issue due to its high morbidity and mortality [1]. The incidence of diabetes in China is increasing rapidly. The prevalence of type 2 diabetes and prediabetes in adults in China reaches 10.9% and 35.7%, respectively [2]. The chronic complications of diabetes are not only the major causes of diabetic disability and death, but also bring heavy economic burdens to patients and society [3–5]. Although the glucose level of prediabetes does not meet the criteria of diabetes, it still causes harmful outcomes and is associated with an increased risk of early nephropathy, small fiber neuropathy, early retinopathy, and cardiovascular disease [6–8]. Therefore, it is of great significance for the early diagnosis and early treatment of patients with prediabetes and diabetes [9]. Nonetheless, the clinical symptoms of prediabetes and early diabetes are not obvious. When patients have significant features of diabetes, including polyuria, polydipsia, polyphagia, and weight loss, and are diagnosed by the gold standard for clinical diabetes diagnosis, namely the oral glucose tolerance test (OGTT), most have begun to develop complications of diabetes, missing the best time for intervention and treatment [10]. Despite the serious outcomes of prediabetes and diabetes, it is regrettable that there is current a high occurrence of a lack of diagnosis. One study showed that the undiagnosed rate of diabetes can reach 60% in China [11]. As a result, there is an urgent need to screen for prediabetes and diabetes in the early subclinical or asymptomatic stage to prevent it from developing to an irreversible stage.

The current diagnosis of prediabetes and diabetes is based on blood glucose or HbA1c, which is invasive and time-consuming, and has a relatively high cost. First, venous blood is obtained from subjects, after which blood glucose is measured using the glucose oxidase method; HbA1c is measured via high-performance liquid chromatography. These methods are accurate and are used as the gold standard to diagnose prediabetes and diabetes, but they require approximately several hours to obtain results, and are not suitable for screening prediabetes and diabetes in a large population. Therefore, a noninvasive, fast, low cost, and safe method should be developed to screen for high-risk populations of diabetes [12]. Effective methods of noninvasive screening for prediabetes and diabetes, including diabetes risk calculator [13], Finnish diabetes risk score [14], artificial neural network [15], TOPICS diabetes screening score [16], Leicester risk assessment score [17], support vector machine model [18], and self-assessment tool [19], are rare at present. These methods usually use multiple factors such as age, body mass index (BMI), waist circumference, height, race/ethnicity, blood pressure, family history, blood lipids, and algorithms, to establish models for predicting prediabetes and diabetes and are inaccurate or complex as assessment systems.

**Fig. 1:**
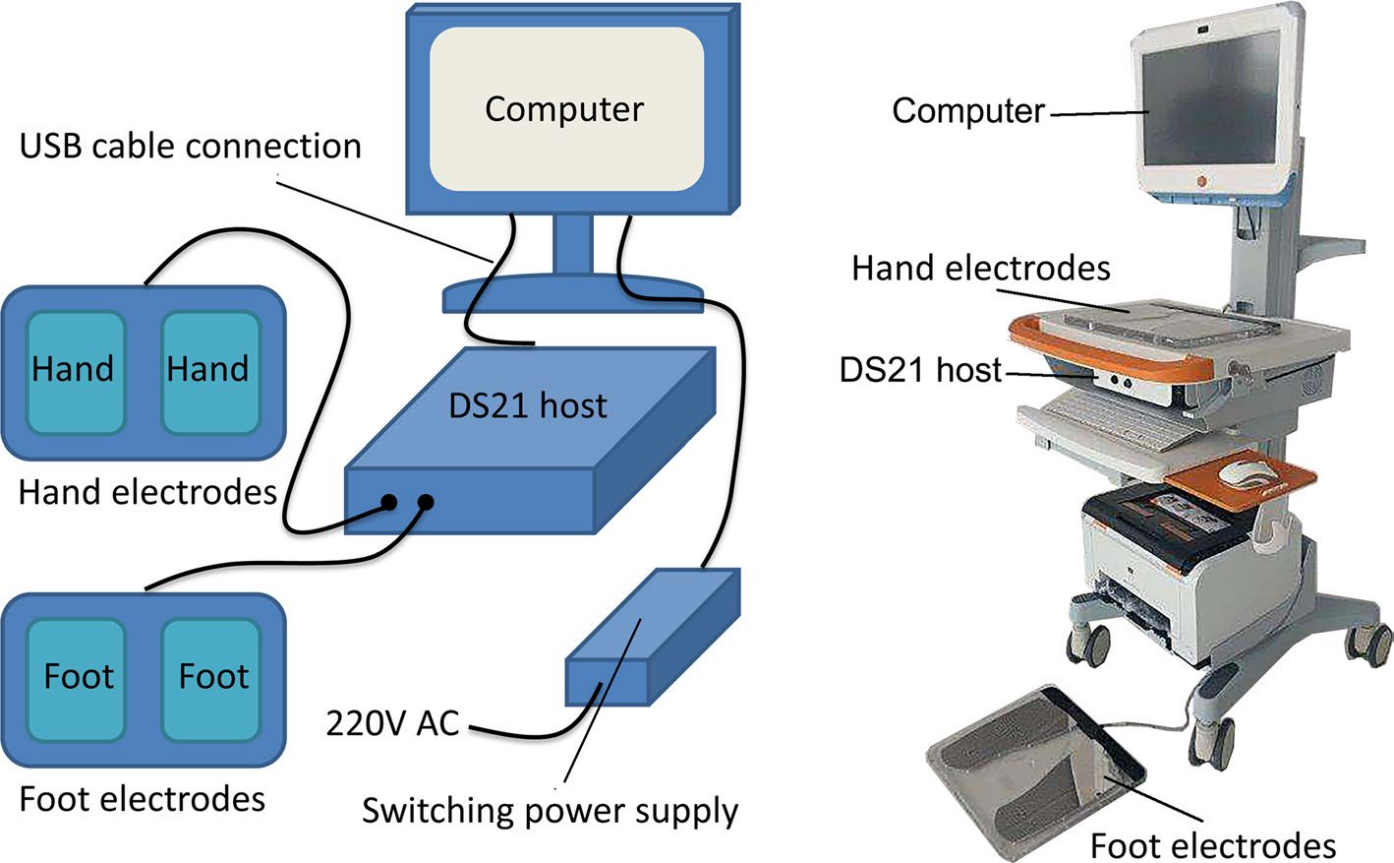
The component of DS21. DS21 was consisted of hand electrodes, foot electrodes, DS21 host, computer and associated software, USB cable connection, and switching power supply

DS21, a new device (Fig. 1), has been developed and applied to screen prediabetes and diabetes based on electrophysiological stimulation feedback [20]. The principle of the electrophysiological stimulation feedback instrument is based on the knowledge that prediabetes and diabetes can cause autonomic neuropathy at an early stage. Neurological damage may occur in the early stages of diabetes, especially the sudomotor nerves of distal limbs, which influences the sudomotor function of sudomotor nerves. Prediabetes and diabetes reduce distal limb sweating, and induce compensatory forehead sweating. The change in sweat gland function is reflected by the concentration of hydrogen ions and chloride ions in sweat. The ionic concentration correlates negatively with the severity of diabetes: sweat has a reduced ionic level when the diabetes is more severe. The electrochemical signal obtained by measuring body parts rich in sweat glands, including the forehead, hands, and feet, can be used in clinical diagnosis and electrophysiological studies. The electrophysiological stimulation feedback instrument measures conductance value by testing the changes in ionic level in limbs sweat, and detects and evaluates diabetes risk based on the conductance value to predict prediabetes and early diabetes [20–24]. Another advantage of the electrophysiological stimulation feedback instrument is that it is fast and easy to operate. The detection takes less than 3 min per subject, and no professional training is needed for the operator [21]. At present, there are two kinds of instruments that measure sweat gland function based on sweat chloride concentrations: EZSCAN and DS21. The key difference between EZSCAN and DS21 is that DS21 has an electrophysiological model of human body under ultra-low direct-current (DC) voltage (DC 1–4 V) condition. The model parameters are optimized through clinical research and the actual measured values are corrected by electrophysiological model calculation and population model, which can more accurately restore the electrochemical reaction process and parameters of human skin. EZSCAN has been widely evaluated [21–29], whereas DS21 has not yet been fully investigated.

In this study, we examined the cut-off value of screening for prediabetes and diabetes, and tested the efficacy and safety of the new noninvasive technology, DS21.

## Results

### Clinical characteristics of the subjects

The demographic characteristics and glycometabolism status of the population are shown in Table 1. There were no significant differences among the three groups in sex or diastolic pressure (all *P *≥ 0.05). Age, BMI, systolic pressure, fasting blood glucose (FBG), 2 h-OGTT plasma glucose (2 h-OGTT PG), and HbA1c showed significant differences among the three groups (*P *< 0.001), the trend of which was diabetes (DM) > impaired glucose regulation (IGR) > normal control (NC) group.

**Table 1:** Characteristics and glycometabolism status of the subjects

	Total (*n* = 939)	NC (*n* = 308)	IGR (*n* = 312)	DM (*n* = 319)	*P* value
Sex (male/female)	439/500	137/171	149/163	153/166	0.621
Age	62 (53, 71)	43 (34, 59)	66 (59, 72)^a^	69 (61, 75)^a,b^	< 0.001
BMI (kg/m^2^)	24.4(23.4, 26.8)	23.1 (21.4, 25.0)	25.0 (22.9, 27.3)^a^	25.7 (23.3, 28.0)^a,b^	< 0.001
Systolic pressure (mmHg)	135 (123, 149)	124 (116, 133)	139 (128, 155)^a^	143 (132, 158)^a,b^	< 0.001
Diastolic pressure (mmHg)	76.8 ± 10.1	77.3 ± 9.9	76.6 ± 11.0^a^	76.7 ± 9.4	0.540
Fasting blood glucose (mmoL/L)	5.7 (5.1, 6.6)	5.3 (5.0, 5.5)	5.8 (5.5, 6.1)^a^	6.7 (6.0, 7.8)^a,b^	< 0.001
2 h-OGTT PG (mmoL/L)	8.4 (5.8, 11.9)	5.3 (4.5, 6.0)	8.6 (8.0, 9.7)^a^	13.7 (11.9, 16.2)^a,b^	< 0.001
HbA1c (%)	5.8 (5.4, 6.4)	5.3 (5.0, 5.5)	5.8 (5.5, 6.1)^a^	6.7 (6.0, 7.8)^a,b^	< 0.001
Conductance value					
Hands–feet	73.9 (58.5, 81.3)	80.9 (75.3, 85.5)	70.5 (56.8, 78.4)^a,c^	65.0 (43.5, 76.9)^a,b,c,d^	< 0.001
Hands	74.2 (59.2, 81.2)	80.7 (75.2, 86.6)	70.4 (57.2, 78.5)^a,c^	65.8 (44.8, 76.8)^a,b,c,e^	< 0.001
Feet	73.7 (57.9, 80.8)	79.8 (74.6, 85.6)	70.5 (55.8, 77.3)^a,c^	63.1 (44.0, 76.6)^a,b,c,e^	< 0.001

*NC* normal control, *IGR* impaired glucose regulation, *DM* diabetes, *BMI* body mass index

^a^*P *< 0.01 vs. NC; ^b^*P *< 0.01 vs. IGR

Adjusting for age and BMI: ^c^*P* < 0.01 vs. NC group; ^d^*P* < 0.05 vs. IGR group; ^e^*P* < 0.01 vs. IGR group

### Comparison of conductance values for hands–feet, hands conductance values, and feet among the NC, IGR, and DM groups

All conductance values for hands–feet, hands, and feet in the NC group were significantly higher than those in the IGR and DM groups, and all conductance values for hands–feet, hands, and feet in the IGR group were significantly higher than those in the DM group (all *P* < 0.01). Previous studies showed that autonomic neuropathy is associated with sex and age [21, 25]. In our study, sex matching was comparable, but the age differed significantly among the three groups. To exclude the effect of age on conductance values, we statistically adjusted for age for further analysis, and all differences among the three groups remained significant after adjustment for age (Table 1).

### Correlation of metabolic parameters with conductance values

Linear correlation analysis was applied to assess the correlation of FBG, 2 h-OGTT PG, HbA1c, age, and BMI with conductance values (Table 2). We found that FBG, 2 h-OGTT PG, HbA1c, age, and BMI were negatively associated with conductance values for hands–feet, hands, and feet (all *P *< 0.01). After adjusting for age and BMI, FBG, 2 h-OGTT PG, and HbA1c were still negatively associated (all *P *< 0.01, Table 2).

**Table 2:** The association of metabolic parameters with conductance values

	Hands–feet	Hands	Feet
FPG	− 0.281^a^	− 0.268^a^	− 0.274^a^
2 h-OGTT PG	− 0.377^a^	− 0.358^a^	− 0.367^a^
HbA1c	− 0.322^a^	− 0.301^a^	− 0.316^a^
Age	− 0.396^a^	− 0.348^a^	− 0.393^a^
BMI	− 0.149^a^	− 0.112^a^	− 0.161^a^
FPG	− 0.181^b^	− 0.182^b^	− 0.171^b^
2 h-OGTT PG	− 0.207^b^	− 0.214^b^	− 0.192^b^
HbA1c	− 0.183^b^	− 0.181^b^	− 0.175^b^

^a^*P *< 0.01; ^b^*P *< 0.01, adjust for age and BMI

### Efficacy of conductance values on screening for impaired glucose regulation or diabetes

**Fig. 2:**
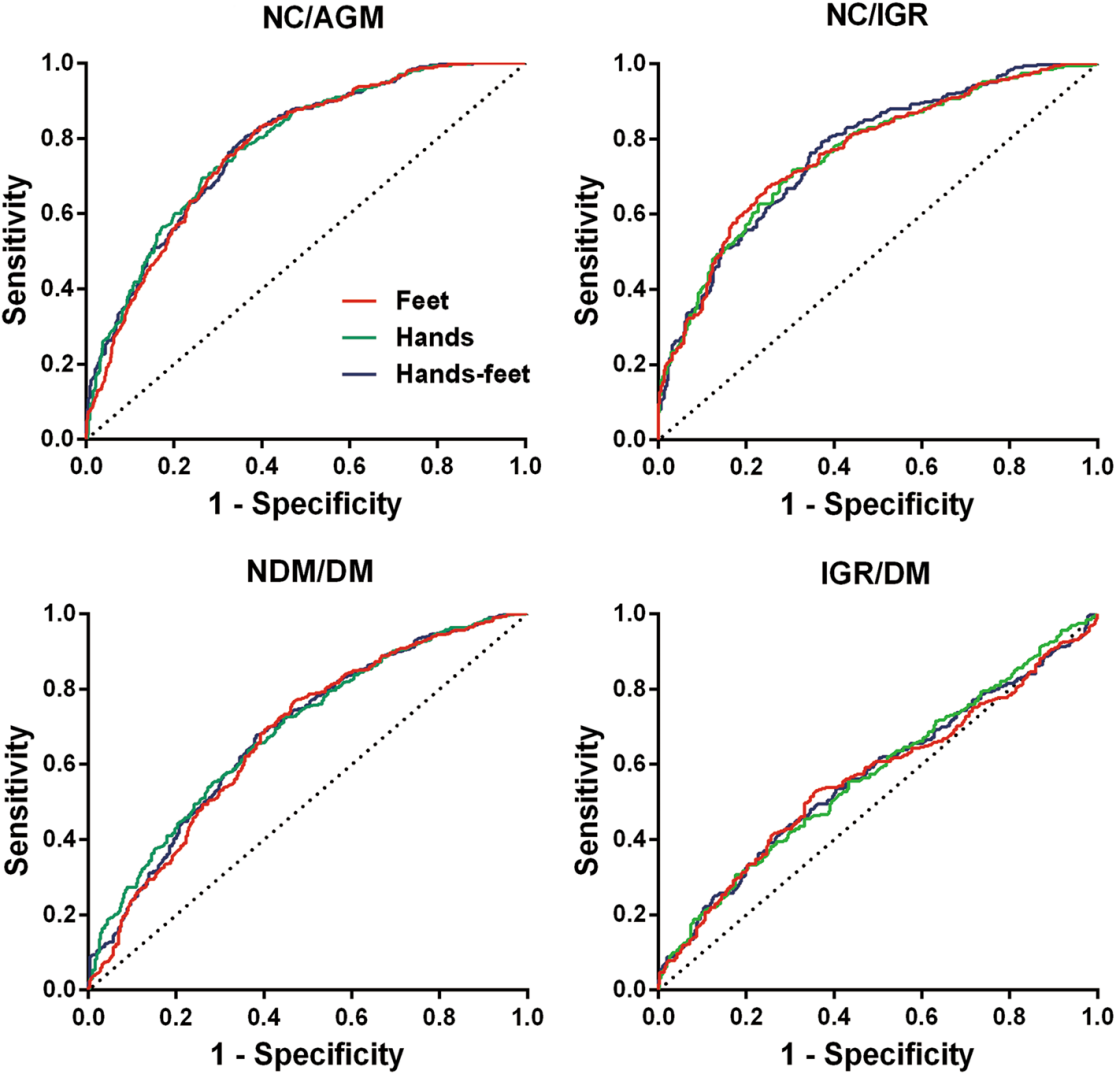
Receiver-operating characteristic (ROC) curves for distinguishing NC/AGM, NC/IGR, NDM/DM, and IGR/DM. NC: normal control; AGM: abnormal glucose metabolism; IGR: impaired glucose regulation; NDM: non-diabetes; DM: diabetes

The area under the ROC curve (AUCROC) was applied to evaluate the efficacy of conductance values on screening for diabetes risk. Due to the higher distinguishing capacity of hand conductance values in most groups except NC/IGR, we recommended hand conductance values as an efficacy value for distinguishing NC/abnormal glucose metabolism (AGM, IGR+DM), NC/IGR, non-diabetes (NDM, NC+IGR)/DM, and IGR/DM for convenience (Fig. 2). When hands conductance values were applied to distinguish NC/AGM, NC/IGR, NDM/DM, and IGR/DM, the AUCROCs were 0.782 [95% CI 0.752, 0.812], 0.761 [95% CI 0.723, 0.798], 0.688 [95% CI 0.653, 0.723], and 0.573 [95% CI 0.528, 0.617], respectively (Table 3). The efficacy of hand conductance values for distinguishing NC/AGM and NC/IGR was stronger than for distinguishing NDM/DM and IGR/DM (Table 3).

**Table 3:** The AUCROC of hands–feet, hands, and feet conductance value for distinguishing NC/AGM, NC/IGR, NDM/DM, and IGR/DM

	NC/AGM		NC/IGR		NDM/DM		IGR/DM	
	AUCROC	*P* value	AUCROC	*P* value	AUCROC	*P* value	AUCROC	*P* value
Hands–feet	0.781 [95% CI 0.752, 0.811]	< 0.001	0.766 [95% CI 0.730, 0.803]	< 0.001	0.683 [95% CI 0.647, 0.719]	< 0.001	0.570 [95% CI 0.525, 0.615]	< 0.001
Hands	0.782 [95% CI 0.752, 0.812]	< 0.001	0.761 [95% CI 0.723, 0.798]	< 0.001	0.688 [95% CI 0.653, 0.723]	< 0.001	0.573 [95% CI 0.528, 0.617]	< 0.001
Feet	0.775 [95% CI 0.745, 0.805]	< 0.001	0.765 [95% CI 0.728, 0.802]	< 0.001	0.675 [95% CI 0.638, 0.712]	< 0.001	0.566 [95% CI 0.521, 0.610]	< 0.001

*NC* normal control, *AGM*, abnormal glucose metabolism (including IGR + DM); *IGR* impaired glucose regulation, *NDM* non-diabetes (including NC + IGR), *DM* diabetes, *CI* confidence interval

### Determination of screening threshold for distinguishing NC/AGM, NC/IGR, NDM/DM, and IGR/DM

We used the hand conductance value to distinguish NC/AGM, NC/IGR, NDM/DM, and IGR/DM, and found that the Youden index was the largest (0.432, 0.413, 0.281, and 0.131) when the value was less than 77.1, 77.1, 68.4, and 51.0, respectively (Table 4). However, screening equipment not only needs accuracy, but also high sensitivity. Therefore, we recommended 75.0 (sensitivity 0.769, specificity 0.660, positive predictive value PPV 0.851, negative predictive value NPV 0.583), 77.1 (sensitivity 0.718, specificity 0.695, PPV 0.702, NPV 0.708), 68.4 (sensitivity 0.726, specificity 0.555, PPV 0.510, NPV 0.760), and 58.1 (sensitivity 0.382, specificity 0.744, PPV 0.602, NPV 0.223) as screening thresholds for distinguishing NC/AGM, NC/IGR, NDM/DM, and IGR/DM, respectively.

**Table 4:** The sensitivity, specificity, positive likelihood ratio, negative likelihood ratio, positive predictive value, negative predictive value, and Youden index under different diagnostic threshold to diagnose NC/AGM, NC/IGR, IGR/DM, and NDM/DM

Cut-off value	Sensitivity	Specificity	PLR	NLR	PPV	NPV	Youden index
NC/AGM							
77.1	0.695	0.737	2.645	0.414	0.796	0.565	0.432
77.0	0.695	0.736	2.629	0.415	0.832	0.563	0.431
76.1	0.724	0.706	2.460	0.391	0.839	0.544	0.430
75.0	0.769	0.660	2.262	0.349	0.851	0.583	0.429
75.8	0.740	0.688	2.375	0.377	0.843	0.537	0.429
NC/IGR							
77.1	0.718	0.695	2.352	0.406	0.702	0.708	0.413
77.0	0.715	0.695	2.342	0.411	0.703	0.706	0.410
77.1	0.718	0.692	2.328	0.408	0.702	0.708	0.410
77.0	0.712	0.695	2.331	0.415	0.703	0.706	0.406
77.2	0.718	0.688	2.303	0.410	0.700	0.707	0.406
NDM/DM							
68.4	0.726	0.555	1.632	0.493	0.510	0.760	0.281
68.5	0.721	0.555	1.621	0.502	0.506	0.759	0.276
68.3	0.726	0.549	1.609	0.499	0.507	0.758	0.275
68.6	0.720	0.555	1.617	0.505	0.504	0.759	0.275
71.5	0.654	0.621	1.724	0.558	0.479	0.770	0.274
IGR/DM							
51.0	0.307	0.824	1.743	0.841	0.641	0.253	0.131
50.8	0.304	0.824	1.725	0.845	0.638	0.253	0.128
51.1	0.307	0.821	1.712	0.844	0.632	0.250	0.128
53.7	0.332	0.795	1.620	0.840	0.624	0.243	0.127
58.1	0.382	0.744	1.492	0.831	0.602	0.223	0.126

*PLR* positive likelihood ratio, *NLR* negative likelihood ratio, *PPV* positive predictive value, *NPV* negative predictive value, *NC* normal control, *AGM* abnormal glucose metabolism (including IGR+DM), *IGR* impaired glucose regulation, *NDM* non-diabetes (including NC+IGR), *DM* diabetes

We also investigated PPV with 90% sensitivity to diagnose NC/AGM, NC/IGR, IGR/DM, and NDM/DM. The PPV reached its highest value (0.903) when 66.0 was applied to distinguish NC/AGM (Table 4).

### Safety evaluation of electrophysiological stimulation feedback instrument measurement

No adverse events or discomfort occurred in the test for 939 cases. The incidence of adverse events was 0%. Therefore, DS21 is safe for prediabetes and diabetes screening.

## Discussion

Blood glucose or HbA1c is not suitable for screening prediabetes and diabetes in large populations due to its invasiveness, time consumption, and relatively high cost. In this study, we found DS21 to be fast, noninvasive, low cost, reliable, and safe, which can be used to screen prediabetes and diabetes in large populations.

The conductance values of hands–feet, hands, and feet measured by DS21 among the three groups (NC, IGR, and DM) were significantly different (all *P* < 0.01), and can be used to distinguish NC/AGM, NC/IGR, NDM/DM, and IGR/DM. Due to the higher distinguishing capacity of hand conductance values in most groups except NC/IGR, we suggested hand conductance values as an efficacy value for distinguishing NC/AGM, NC/IGR, NDM/DM, and IGR/DM. The diagnostic accuracy of hand conductance values in distinguishing NC/AGM and NC/IGR was moderate, though the PPV was highest when a conductance value of 66.0 was applied to distinguish NC/AGM.

Previous studies have shown that EZSCAN is an effective method to predict prediabetes and diabetes. Studies reported a low sensitivity (29%) of FBG for detecting DM when 7.0 mmol/L was used as a cut-off value [22]. However, the predictability of EZSCAN for prediabetes is even higher than that of FBG, 2 h-OGTT glucose, 1h-OGTT glucose, and HbA1c. Additionally, a cross-sectional study including 270 undiagnosed patients with a high risk of glucose metabolism disorders revealed a cut-off value of EZSCAN for the detection of impaired glucose tolerance (IGT) of 37% (sensitivity, 82%; specificity, 62%; AUCROC 0.778), with a cut-off point for NDM of 50% (sensitivity, 53%; specificity, 59%; AUC, 0.528) [26]. Another trial including 1414 patients reported that the AUCROC for EZSCAN to predict prediabetes and diabetes was 0.65 and 0.73, respectively. The sensitivity for EZSCAN in detecting prediabetes and diabetes was 69% and 73%, with a specificity of 56% and 70%, respectively [21]. In addition, a study enrolling 212 Asian Indian subjects found that when 50% was used as the threshold, the sensitivity of EZSCAN for the diagnosis of DM, IGT, and normal glucose tolerance with metabolic syndrome was 75%, 70% and 84%, respectively [22]. Another study including 195 subjects showed that EZSCAN had a sensitivity of 85% and a specificity of 64% for detecting DM with a diabetes index at a threshold > 40% [29]. In the current study, DS21 had a similar or even better efficacy for prediabetes (sensitivity 71.8%, specificity 69.5%) and diabetes (sensitivity 72.6%, specificity 55.5%) screening than EZSCAN.

The efficacy of the conductance value for distinguishing NC/AGM and NC/IGR was stronger than the efficacy of the conductance value for distinguishing NDM/DM and IGR/DM. Additionally, FBG, 2 h-OGTT PG, and HbA1c were negatively associated with conductance values of hands–feet, hands, and feet. The reason may be associated with great changes in sweat gland secretion in populations with normal or abnormal glucose metabolism. Furthermore, sweat gland secretion in people with diabetes may not change greatly compared with prediabetes, which might result in this phenomenon. Regardless, the precise mechanism is unknown and should be investigated in the future. Chen et al. reported the same in a Chinese population using EZSCAN [26], while other studies showed opposite results in Asian Indian populations and Mexican populations [21, 22]. This vague result may be due to the different ethnicities involved.

Due to the higher distinguishing capacity of hand conductance values in most groups except NC/IGR, we suggested hand conductance values as an efficacy value for distinguishing NC/AGM, NC/IGR, NDM/DM, and IGR/DM. We recommended 75.0 (sensitivity 0.769, specificity 0.660), 77.1 (sensitivity 0.718, specificity 0.695), 68.4 (sensitivity 0.726, specificity 0.555), and 58.1 (sensitivity 0.382, specificity 0.744) as the screening thresholds for distinguishing NC/AGM, NC/IGR, NDM/DM, and IGR/DM, respectively. We also recommended 66.0 for distinguishing NC/AGM due to the high sensitivity and high PPV.

Regarding the safety of DS21, there was no adverse event in the test in 939 patients, indicating that this device is safe and suitable for screening prediabetes and diabetes.

## Conclusions

Screening for prediabetes and asymptomatic diabetes for early detection of diabetes is important to prevent them from developing to an irreversible stage. In this study, we found that a new device, DS21, is fast, noninvasive, low cost, reliable, and safe and a potential candidate to screen for prediabetes and diabetes. Hand conductance values of 75.0 (sensitivity 0.769, specificity 0.660), 77.1 (sensitivity 0.718, specificity 0.695), 68.4 (sensitivity 0.726, specificity 0.555), and 58.1 (sensitivity 0.382, specificity 0.744) are recommended as the screening thresholds for distinguishing NC/AGM, NC/IGR, NDM/DM, and IGR/DM, respectively. A hand conductance value of 66.0 is also recommended to distinguish NC/AGM due to high sensitivity and high PPV. However, subsequent validation in a large population is still needed.

## Methods

### Study subjects

The present study included 939 participants from the Shanghai Changfeng community (500 women and 439 men, mean age 59.8 ± 15.4 years). The participants were divided into a normal glucose metabolism  group (normal  control,  NC, *n* = 308, 171 women and 137 men), an impaired glucose regulation group (IGR, *n* = 312, 163 women and 149 men), and a diabetes group (DM, *n* = 319, 166 women and 153 men) based on OGTT and diabetic history (1999 WHO diabetes diagnostic criteria). The inclusion criteria for the participants were: i) age > 20 years and ii) NC, IGR, or diabetes diagnosed by OGTT. Patients with type 2 diabetes who had already been diagnosed were allocated into the diabetes group regardless of the OGTT result. The exclusion criteria for participants were as follows: (i) a transient increase in glycemia during infection and stress, type 1 diabetes, gestational diabetes, or other specific types of diabetes; (ii) electrical implantable device; (iii) epilepsy/seizures; (iv) severe organic or systemic diseases; (v) pregnancy; (vi) breast-feeding women or suspected pregnancy; (vii) malignancy; (viii) structural anomaly of the examination position; (ix) participants who were afraid of the examination or were excessively nervous; and (x) participants who were incapable of expressing their feelings. All protocols were in accordance with the Helsinki Declaration of 1975 and approved by the ethics committee of Zhongshan Hospital, Fudan University, and each subject provided written informed consent. The approval numbers of this study are 2014-34 and 2017-012R.

### Anthropometric and laboratory measurements

All subjects underwent routine anthropometric measurements, serum biochemical examinations, and history collections. Standing height, waistline, and body weight were measured without shoes and outer clothing, and BMI was calculated as weight (kg) divided by standing height squared (m^2^). Blood samples were collected after a fasting period of at least 12 h. FBG and 2 h-OGTT PG were measured through the glucose oxidase method And HbA1c via high-performance liquid chromatography.

### DS21 measurements

DS21 is the first-generation product independently developed by Shanghai Zhongjia Hengtai Medical Technology Co., Ltd., which passes the test of the Chinese State Food and Drug Administration (No. ZC2017-265)  and is used to test the electric reaction of skin and evaluate the glucose metabolism of human body through conductivity. All participants underwent testing by DS21. During the test, the participant put his/her hands and feet on two large-area nickel electrodes, and stood for 2 min. A DC incremental voltage ≤ 4 V was applied between the two nickel electrodes. The voltage decreased by 0.2 V per second from 3.8 to 1.0 V. Two nickel electrodes were alternately used as the anode and cathode. The conductance value, which has a positive correlation with the chloride ions concentration of sweat, was determined by chronoamperometry. The conductance values were analyzed based on the hands–feet conductance values, hands conductance values, and feet conductance values.

### Safety evaluation

The adverse event of DS21 test was recorded directly after test, and the incidence of adverse events was calculated. Adverse event was defined as (i) headache; (ii) nausea and vomiting; (iii) electrified sensation; and (iv) skin damage. The incidence of adverse events = number of adverse event cases/total number of cases × 100%. The incidence of adverse reactions ≤ 1% was applied as the safety evaluation criterion.

### Statistical analyses

All statistical analyses were performed using SPSS software version 20.0 (SPSS, Chicago, IL, USA). For continuous variables, the results are shown as mean ± standard deviation (SD) or medians with the interquartile range. For categorical variables, the results are shown as percentages (%). Skewed variables were log transformed to approximate normal distribution before analysis. Intergroup comparisons of continuous data were performed using ANOVA, whereas intergroup comparisons of categorical variables were performed using the Chi-squared test. Correlations of conductance value with FBG, 2 h-OGTT PG, and HbA1c were investigated using linear correlation analysis. The area under the receiver-operating characteristic curve (AUCROC) was applied to evaluate the diagnostic efficacy of the conductance value. AUCROC  ≤ 0.5 was regarded as having no significance in diagnosis; 0.5 < AUCROC ≤ 0.7 was regarded as having low diagnostic accuracy; 0.7 < AUCROC ≤ 0.9 was regarded as having moderate diagnostic accuracy; 0.9 < AUCROC < 1.0 was regarded as having high diagnostic accuracy; AUCROC = 1.0 was regarded as ideal diagnostic accuracy.

## Data Availability

The datasets used and/or analyzed during the current study are available from the leading corresponding authors H.B. (email: bianhuaer@126.com; zhongshan_bh@126.com) on reasonable request.

## Abbreviations

OGTT: Oral glucose tolerance test
BMI: Body mass index
IGT: Impaired glucose tolerance
NC: Normal control
IGR: Impaired glucose regulation
DM: Diabetes
FBG: Fasting blood glucose
2 h-OGTT PG: 2 h-oral OGTT plasma glucose
DC: Direct current
SD: Standard deviation
AUCROC: Area under the ROC curve
